# Modulation of Biliary Cancer Chemo‐Resistance Through MicroRNA‐Mediated Rewiring of the Expansion of CD133+ Cells

**DOI:** 10.1002/hep.31094

**Published:** 2020-09-10

**Authors:** Pietro Carotenuto, Somaieh Hedayat, Matteo Fassan, Vincenzo Cardinale, Andrea Lampis, Vincenza Guzzardo, Caterina Vicentini, Aldo Scarpa, Luciano Cascione, Daniele Costantini, Guido Carpino, Domenico Alvaro, Michele Ghidini, Francesco Trevisani, Robert Te Poele, Massimiliano Salati, Sofia Ventura, Georgios Vlachogiannis, Jens C. Hahne, Luke Boulter, Stuart J. Forbes, Rachel V. Guest, Umberto Cillo, Ian Said‐Huntingford, Ruwaida Begum, Elizabeth Smyth, Vasiliki Michalarea, David Cunningham, Lorenza Rimassa, Armando Santoro, Massimo Roncalli, Vladimir Kirkin, Paul Clarke, Paul Workman, Nicola Valeri, Chiara Braconi

**Affiliations:** ^1^ Division of Cancer Therapeutics Institute of Cancer Research London United Kingdom; ^2^ Telethon Institute of Genetics and Medicine Pozzuoli Italy; ^3^ Division of Molecular Pathology Institute of Cancer Research London United Kingdom; ^4^ Department of Medicine Surgical Pathology Unit University of Padua Padua Italy; ^5^ Department of Medico‐Surgical Sciences and Biotechnologies Sapienza University of Rome Latina Italy; ^6^ Department of Pathology University of Verona Verona Italy; ^7^ Bioinformatics Core Unit Institute of Oncology Research Bellinzona Switzerland; ^8^ Department of Movement, Human and Health Sciences University of Rome Foro Italico Rome Italy; ^9^ Department of Translational and Precision Medicine Sapienza University Rome Italy; ^10^ Medical Oncology and Hematology Unit Humanitas Cancer Center Humanitas Clinical and Research Center‐IRCCS Rozzano Italy; ^11^ MRC Human Genetics Unit Institute of Genetics and Molecular Medicine Edinburgh United Kingdom; ^12^ Center for Regenerative Medicine University of Edinburgh Edinburgh United Kingdom; ^13^ The Royal Marsden NHS Trust Surrey and London Sutton United Kingdom; ^14^ Department of Biomedical Sciences Humanitas University Pieve Emanuele Italy; ^15^ Department of Pathology Humanitas Research Hospital & Hunimed University Rozzano Italy; ^16^ The Institute of Cancer Sciences University of Glasgow Glasgow United Kingdom

## Abstract

**Background and Aims:**

Changes in single microRNA (miRNA) expression have been associated with chemo‐resistance in biliary tract cancers (BTCs). However, a global assessment of the dynamic role of the microRNome has never been performed to identify potential therapeutic targets that are functionally relevant in the BTC cell response to chemotherapy.

**Approach and Results:**

High‐throughput screening (HTS) of 997 locked nucleic acid miRNA inhibitors was performed in six cholangiocarcinoma cell lines treated with cisplatin and gemcitabine (CG) seeking changes in cell viability. Validation experiments were performed with mirVana probes. MicroRNA and gene expression was assessed by TaqMan assay, RNA‐sequencing, and *in situ* hybridization in four independent cohorts of human BTCs. Knockout of microRNA was achieved by CRISPR‐CAS9 in CCLP cells (MIR1249KO) and tested for effects on chemotherapy sensitivity *in vitro* and *in vivo*. HTS revealed that MIR1249 inhibition enhanced chemotherapy sensitivity across all cell lines. MIR1249 expression was increased in 41% of cases in human BTCs. In validation experiments, MIR1249 inhibition did not alter cell viability in untreated or dimethyl sulfoxide–treated cells; however, it did increase the CG effect. MIR1249 expression was increased in CD133+ biliary cancer cells freshly isolated from the stem cell niche of human BTCs as well as in CD133+ chemo‐resistant CCLP cells. MIR1249 modulated the chemotherapy‐induced enrichment of CD133+ cells by controlling their clonal expansion through the Wnt‐regulator FZD8. MIR1249KO cells had impaired expansion of the CD133+ subclone and its enrichment after chemotherapy, reduced expression of cancer stem cell markers, and increased chemosensitivity. MIR1249KO xenograft BTC models showed tumor shrinkage after exposure to weekly CG, whereas wild‐type models showed only stable disease over treatment.

**Conclusions:**

MIR1249 mediates resistance to CG in BTCs and may be tested as a target for therapeutics.

**Abbreviations**
ABC: advanced biliary cancer
BTC: biliary tract cancer
CCA: cholangiocarcinoma
CG: cisplatin and gemcitabine
CRISPR‐CAS9: CRISPR‐associated protein‐9 nuclease
CSC: cancer stem cell
CTRL: control
DMSO: dimethyl sulfoxide
FACS: fluorescence‐activated cell sorting
FZD8: frizzled class receptor 8
gRNA: single‐guide RNA
HTS: high‐throughput screening
ISH: *in situ* hybridization
KO: knockout
LNA: locked nucleic acid
miRNA: microRNA
MIR: microRNA
TCGA: The Cancer Genome Atlas
WT: wild type

Biliary tract cancers (BTCs) include cholangiocarcinoma (CCA) and gallbladder cancer, and their incidence is increasing worldwide.^1^, ^2^, ^3^, ^4^ Lack of effective radical treatments and rapid failure of palliative ones highlight the need for a better understanding of BTC biology and mechanisms of response to treatment.^2^, ^5^ Eighty percent of patients with BTCs present at an advanced stage, when treatment options are limited to chemotherapy with cisplatin and gemcitabine (CG).^5^ Only 11% of patients gain a long‐term benefit from chemotherapy, while primary resistance is detected in 20% of patients.^6^ Most patients develop secondary resistance after an initial response or stabilization of the disease, which is responsible for a global median overall survival shorter than 12 months. Several mechanisms of chemo‐resistance may act synergistically and drive cancer cells to escape biochemical inhibition or cell death caused by chemotherapy.^7^ Acquisition of stemness features in cancer cells appears to be a driver of resistance that is common with other tumor types.^8^, ^9^

MicroRNAs (miRNAs) are small noncoding RNAs controlling mRNA expression.^10^ We and others demonstrated that miRNAs are aberrantly expressed in BTCs and promote biliary carcinogenesis.^11^, ^12^, ^13^, ^14^, ^15^, ^16^, ^17^, ^18^, ^19^ Despite growing evidence that links single miRNAs with chemo‐resistance, no comprehensive genome‐wide approach has been undertaken to date to assess the functional role of miRNAs in the cellular dynamics involved in drug response in BTCs. It is known that chemo‐resistance is a peculiar feature of BTCs, which is responsible for the poor prognosis of these patients. Thus, in this study we have investigated the functional role of microRNA inhibitors in mediating drug response in chemotherapy‐treated BTC cells, using a high‐throughput approach that investigates the inducible role of miRNAs in response to cancer drugs.

## Experimental Procedures

### Human Tissues

The human BTC tissues were collected under approval of the Ethical Committee for Clinical Research at three independent institutions: the Humanitas Research Hospital (#21072014; cohort 1), the Royal Marsden Hospital (CCR 4415; cohort 2), and the University Hospital of Padua (#0010416; cohort 3). The study protocols conformed to the ethical guidelines of the 1975 Declaration of Helsinki, as per ethical approval given by the institutional review board. Formalin‐fixed paraffin‐embedded tissues were retrieved and RNA was extracted from the tumor and the matched nontumor component after microscopic dissection using the Ambion RecoverAll kit (Thermo Fisher Scientific, Waltham, MA). Relapse‐free survival was used as an endpoint of the study. Disease recurrence was defined as the presence of imaging‐proven disease.

### High‐throughput Screening

A human locked nucleic acid (LNA) miRNA inhibitor library (miRCURY LNA version 3; #190102‐3) was purchased from Exiqon (Life Technologies, Paisley, United Kingdom). The library was distributed across fifteen 96‐well plates (Greiner Bio‐One, Frickenhausen, Germany) in a volume of 5 uL in each well. Each plate included two negative controls (LNA negative A and LNA negative B from Exiqon) and positive controls (AllStars Hs positive cell death phenotype control, SI04381048; Qiagen, Manchester, United Kingdom). Fifteen microliters of transfecting solution with medium and Hiperfect (PN301705; Qiagen) was added to each well. Thirty microliters of cell solution was then added to each well to have a final concentration of 10,000 cells and 50 nM of miRNA inhibitors. A column with no cells (×8) was added in one plate. Forty‐eight hours later, 50 uL of a combination of cisplatin (232120; Sigma‐Aldrich, Gillingham, United Kingdom) and gemcitabine (1288463‐200MG; Sigma‐Aldrich) diluted in medium were added. Cisplatin was dissolved in sterile phosphate‐buffered saline and stocked at a concentration of 1 mg/mL (3 mM). Gemcitabine was diluted in dimethyl sulfoxide (DMSO) and stocked at a concentration of 10 mg/mL (30 mM). Both stocks were then diluted in medium to achieve a final solution that would always contain less than 0.001% of DMSO. Cell viability was measured 72 hours later by CellTiter‐Blue Assay (Promega, Madison, WI). The cell‐viability measurement from each hit was normalized to that of the averaged negative controls across the respective plate. Each cell line was tested in triplicate. Statistical significance (*P ≤* 0.05) was determined by two‐sided Student *t* test across three replicates.

### MIR1249‐Knockout Generation Through CRISPR‐CAS9

CCLP‐1 cells were transfected using Lipofectamine 3000 reagent (Thermo Fisher Scientific), with the CRISPR vector pCAS‐Guide‐EF1a‐GFP CRISPR Vector (GE100018; OriGene Technologies, Inc., Rockville, MD) expressing single‐guide RNAs (gRNAs) containing the inserted target sequence for miR1249. Target sequences of the gRNA were as follows: gRNA3 5′ CGTCGGTCGTGGTAGATAGG 3′; gRNA4 5′ AATCTCGACCGGACCTCGAC 3′). Forty‐eight hours later, green fluorescent protein–positive cells were sorted with a FACSAria‐II (BD Biosciences, San Jose, CA) and maintained in culture. Genome editing was verified at day 17 using the Indel identification kit (Clontech Biotec, Mountain View, CA). Cells were enriched for the edited clones by performing serial dilution. Final assessment of the successful genome editing was performed by sequencing and real‐time PCR.

### Statistical Analyses

Statistical analyses were performed by GraphPad Prism 6 (La Jolla, CA). Results are expressed as mean ± SD, unless indicated otherwise. Groups were compared with either a two‐tailed Student *t* test (for analysis of two groups) or using one‐way analysis of variance to compare multiple groups. Significance was accepted when *P* was less than 0.05.

CCLP‐1 BTC XENOGRTAFT MODEL. BTC xenograft tumours (WT N:20; MIR1249KO N:20) were established subcutaneously in 6‐7 weeks female NOD‐scid IL2Rgnull mice (Charles River Laboratories, Wilmington, MA, USA). The study was performed in accordance with UK Home Office regulations under the Animals Scientific Procedures Act 1986 and in accordance with UK National Cancer Research Institute guidelines and the ARRIVE guidelines. Animals were housed in specific pathogen‐free rooms in autoclaved, aseptic microisolator cages with a maximum of five animals per cage. Please see supplementary material for more information.

## Results

### High‐throughput Functional Studies and Characterization of Human Cancer Tissues Identified MIR1249 Inhibition as a Clinically Relevant Strategy to Increase Chemo‐sensitivity in Human BTCs

**Figure 1:**
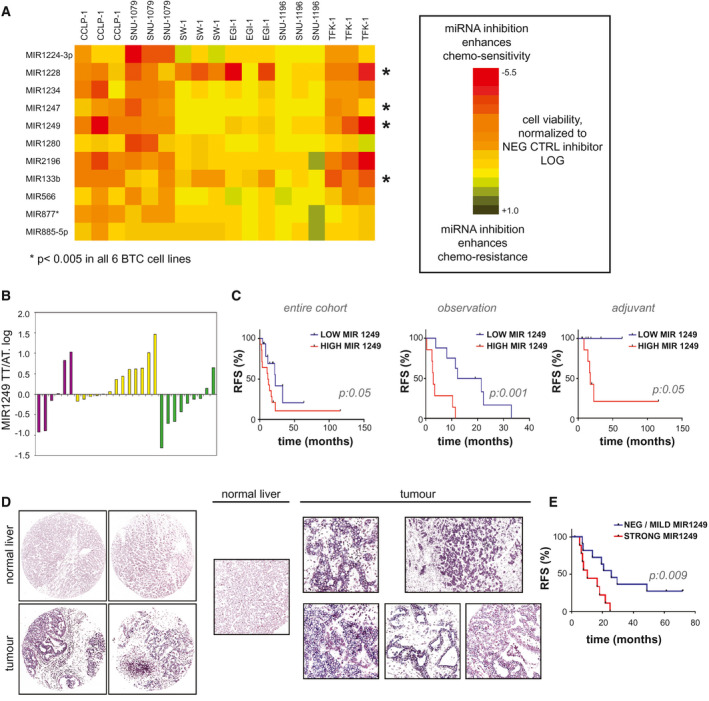
MIR1249 represents a clinically significant candidate for therapeutics based on *in vitro* HTS data and expression profiles of human BTC samples. (A) HTS technologies were applied to six cell lines in triplicate. Cells were reverse‐transfected with LNA miRNA inhibitors for 48 hours, followed by treatment with CG chemotherapy (see also Supporting Fig. S1). After 72 hours, cell viability was assessed by Cell‐Titer blue assay. Each square indicates the logarithmic value of the mean of cell viability normalized to averaged negative controls (n = 3), and the color code indicates the degree of change in cell viability. Here we show miRNA inhibitors that were significant (*P* < 0.05) in enhancing chemo‐sensitivity across all intrahepatic CCA or across all extrahepatic CCA (*P* < 0.05). *MicroRNA inhibitors that were significant across all six cell lines (see also Supporting Table S1). (B) MIR1249 expression was assessed by TaqMan assay in the tumor tissue (TT) and adjacent tissue (AT) of 29 human clinically annotated BTC samples (cohort 1) (see also Supporting Table S2). Insufficient RNA quality was achieved for the adjacent tissue of one case; thus, the data for 28 cases are shown. Bars represent the mean values of two technical replicates for each patient. Purple, yellow, and green bars indicate intrahepatic CCA, extrahepatic CCA and gallbladder cancer, respectively. (C) Kaplan‐Meier analysis was used to correlate relapse‐free survival with MIR1249 tumor tissue expression. Cases were classified into low and high MIR1249 expression according to the median value. (D) MIR1249 expression was assessed by ISH in 28 formalin‐fixed paraffin‐embedded human BTC tissues (cohort 2). Representative pictures are shown (see also Supporting Table S3). Original magnifications: ×10 (left) and 20 (right). (E) Kaplan‐Meier curves in cohort 2 with respect to MIR1249 expression (strong: ISH score 2+ or greater; negative/mild: ISH score 0 or 1+). Abbreviations: AT, adjacent tissue; RFS, relapse‐free survival; TT, tumor tissue.

High‐throughput screening (HTS) technologies were applied to screen a panel of six CG‐treated BTC cell lines against a library of LNA miRNA inhibitors. The 50% growth inhibitory concentration for CG was derived for each cell line to define the concentration at which the combination of CG could induce cytotoxicity without reducing cell viability by more than 50%, to enable identification of sensitizers (Supporting Fig. S1). HTS was run in triplicate in each cell line (Supporting Table S1). Inhibition of MIR148a and members of the let‐7 family reduced sensitivity in a number of cell lines, in line with published literature.^13^, ^20^ Eleven miRNA‐inhibitors acted as sensitizers in all intrahepatic CCA cells, and four in all extrahepatic CCA cells (*P* < 0.05) (Fig. 1A). Inhibitors of MIR1249, MIR133b, MIR1247, and MIR1228 decreased cell viability across all cell lines in comparison to control (CTRL) inhibitors. The tissue expression of these four short‐listed miRNAs was determined in human BTCs by TaqMan assay to investigate the clinical relevance of these candidates. MIR133b, MIR1247, and MIR1228 expression was not increased in the tumor tissue in comparison to matched adjacent nontumor tissue (cohort 1, n = 29) (Supporting Fig. S2A). Conversely, MIR1249 was overexpressed in the tumor compartment in comparison to paired nontumor tissue in 32% of cases (Fig. 1B). Interestingly, when the cohort was split according to median MIR1249 tumor expression, cases with high expression were associated with worse prognosis independently of adjuvant chemotherapy (Fig. 1C and Supporting Table S2). At multivariate analysis (considering T stage, N stage, adjuvant chemotherapy and MIR1249 tumor expression), adjuvant treatment (hazard ratio [HR] 0.70; *P* < 0.001) and MIR1249 expression (HR 0.65; p: 0.004) maintained an independent prognostic value. An increase of MIR1249 expression by TaqMan assay was observed in 53% of the cases of a separate cohort (cohort 2, n = 28) (Supporting Fig. S2B). When cohorts 1 and 2 were pooled together, 41% of cases showed increased MIR1249 expression in the tumor. RNA‐seq data confirmed overexpression of MIR1249 in the tumor tissue in comparison to paired normal tissue in 55% of cases (The Cancer Genome Atlas [TCGA] cohort,^21^ n = 9) (Supporting Fig. S2C). Kaplan‐Meier analysis of the whole TCGA cohort showed that tumor MIR1249 expression was again associated with progression‐free interval (Supporting Fig. S2D). When assessed by *in situ* hybridization (ISH), MIR1249 was strongly positive in 53% of the tumor cases and was statistically associated with lower survival outcome (cohort 3, n = 28) (Fig. 1D‐E and Supporting Table S3).

### Validation Functional Studies Identified the Involvement of MIR1249 in Driving a Chemotherapy‐Specific Reactive Response in Cancer Cells

**Figure 2:**
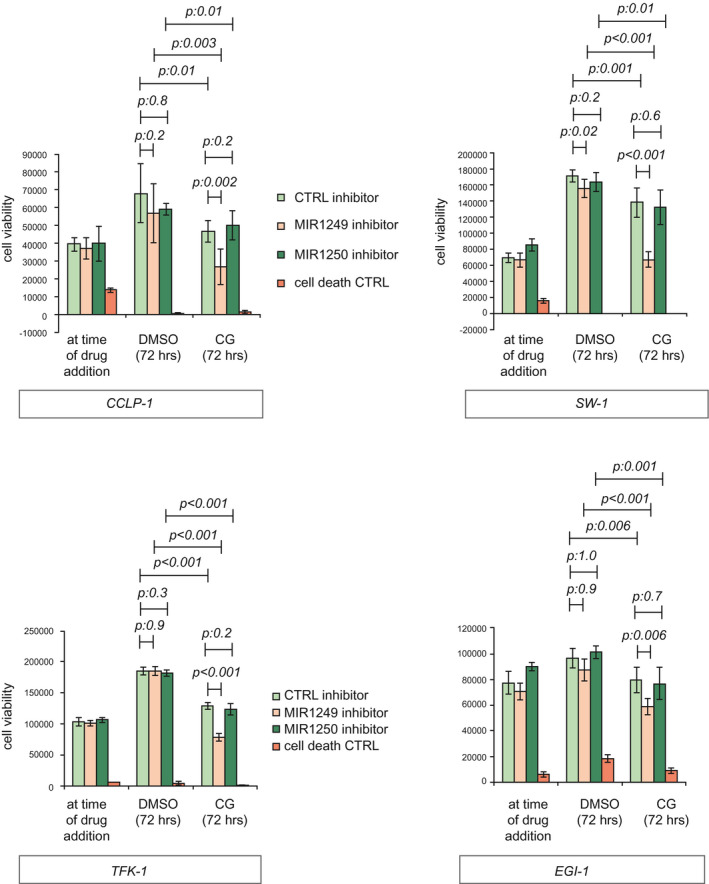
MIR1249 inhibition decreases cell viability in chemotherapy‐treated cells only. MIR1249 inhibition was achieved using mirVana inhibitory probes (Thermo Fisher Scientific). Cells were plated in 96‐well plates and transfected with the indicated probes for 48 hours before being treated with DMSO or CG for a further 72 hours. Cell death CTRL was used as a positive control. Cell viability was assessed by CellTiter Blue assay (Promega, Madison, WI). Bars indicate the mean of six independent experiments ± SD.

**Figure 3:**
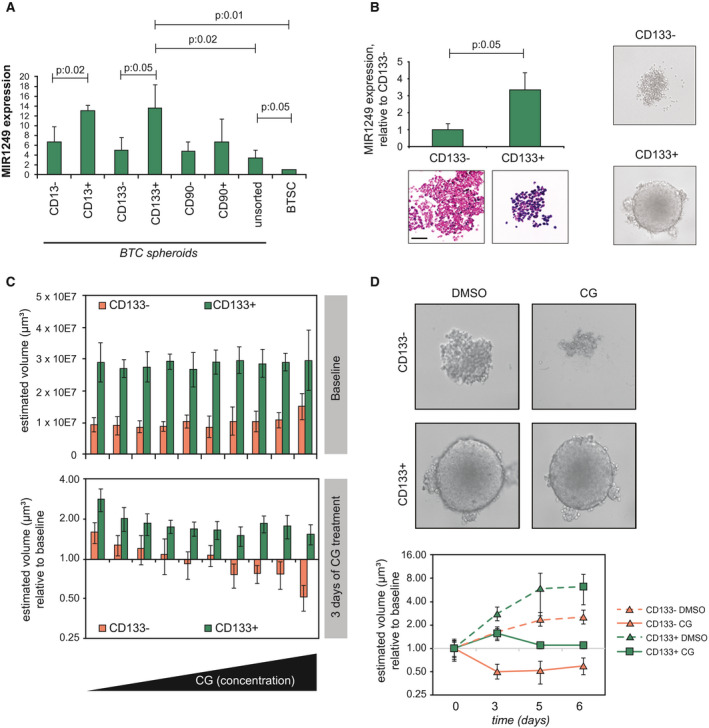
Human CD133+ BTC cells hold high MIR1249 levels and are highly chemo‐resistant. (A) CSC niche of human BTC samples was identified. Cells were then extracted, sorted by cell surface markers, and cultured in 3D spheroids. MIR1249 expression was assessed by TaqMan assay. Bars indicate three independent replicates ± SD. (B) CCLP‐1 cells were sorted by FACS for CD133 surface expression. MIR1249 was assessed both by TaqMan (bars indicate the mean of three independent experiments ± SD) and by ISH (representative pictures are shown; scale bar: 100 μM). (C) When cultured in ultralow attachment plates, CD133+ cells formed large and defined 3D spheroids conversely to CD133‐ cells. (C) CCLP‐1 cells were sorted for CD133 surface expression and cultured in ultralow attachment plates. After 5 days, baseline imaging of spheroids by Celigo showed reproducible spheroid formation across the plates, even though CD133‐ spheroids were smaller. After 3 days of CG treatment (scalar concentrations up to 3 μM cisplatin and 30 nM gemcitabine), CD133‐ spheroids shrank in volume, whereas CD133+ spheroids did not, even at the highest CG concentration. Bars represent the mean of six replicates ± SD. (D) Representative pictures of spheroids with and without CG treatment. Spheroids were monitored up to 6 days. The volume of CD133‐ spheroids decreased from baseline, whereas the volume of CD133+ spheroids was stabilized (see also Supporting Fig. S2). Abbreviation: BTSC, biliary tract stem cells.

**Figure 4:**
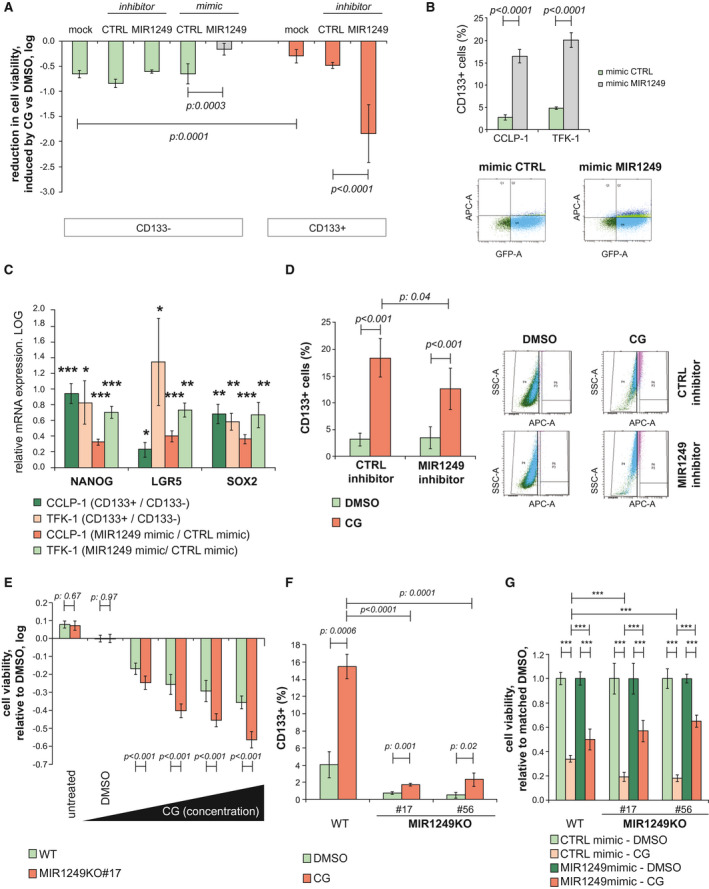
MIR1249 inhibition prevents chemotherapy‐induced enrichment of the CD133+ subclone. (A) CCLP‐1 cells were transfected with indicated probes after being sorted for CD133 surface expression. Mock indicates the absence of any probe. Chemotherapy was added 48 hours after transfection, and cell viability was read 72 hours later by CellTiter Blue assay. Bars indicate the mean of six independent experiments ± SD. (B) BTC cells were transfected and assessed for CD133+ by fluorescence‐activated cell sorting (FACS). The population of CD133+ cells increased with enforced expression of MIR1249. Bars indicate the mean of three independent experiments ± SD. Representative pictures of the FACS analysis are shown; CD133+ cells are identified by the double positivity of allophycocyanin axis and green fluorescent protein axis (right upper quadrant). (C) BTC cells were sorted for CD133 and collected for RNA extraction. Bars represent the log value of the indicated ratio with the relative control (positive values above the *x* axis indicate an increase in expression versus relative control). All markers are increased in CD133+ (vs. CD133‐) cells and after transfection of unsorted cells with MIR1249 mimic (vs. mimic control). Bars indicate the mean of at least four independent replicates ± SEM. *P < 0.05; **P < 0.01; ***P < 0.001 versus relative control. (D) CCLP‐1 cells were treated with DMSO or CG chemotherapy after transfection with CTRL or MIR1249 inhibitor and assessed for CD133+ by FACS. CG induced enrichment of CD133+ cells, and this enrichment was reduced in the case of transfection with MIR1249 inhibitor. Bars represent the mean of three independent experiments ± SD. Representative pictures of FACS analysis are shown on the right. Pink dots represent CD133+ cells. (E) WT and MIR1249KO CCLP‐1 cells were treated with DMSO and CG chemotherapy at scalar doses up to 5 M cisplatin and 50 nM gemcitabine. Bars represent the log of the ratio between CG‐treated and DMSO‐treated cells. Bars indicate the mean of six independent experiments ± SD. (F) WT and two clones of MIR1249KO cells were treated with DMSO and CG chemotherapy and assessed for CD133 expression by FACS. Bars represent the mean of three independent experiments ± SD. (G) Cells were transfected with CTRL mimic or MIR1249 mimic and treated with DMSO and CG chemotherapy. Bars represent the mean of six independent experiments ± SD. ***P < 0.001 (see also Supporting Fig. S3). Abbreviations: APC‐A, allophycocyanin axis; GFP‐A, green fluorescent protein axis; LGR5, leucine‐rich repeat‐containing G protein‐coupled receptor 5; SOX2, SRY (sex determining region Y)‐box 2.

On the basis of the clinical and biological relevance of these data, MIR1249 was selected as a candidate for further studies. MIR1250 inhibitor (which exhibited no effect on any of the cells in the HTS) was included in the validation phase as a negative control, along with scrambled control (Supporting Fig. S2E). Using alternative probes for miRNA inhibition, the ability of MIR1249 inhibitor to enhance BTC cell response to CG chemotherapy was validated. Interestingly, we did not observe a cytotoxic effect for MIR1249 inhibitor in the absence of chemotherapy treatment (Fig. 2), suggesting that MIR1249 interferes with a chemotherapy‐specific response. Indeed, MIR1249 expression was increased as a response to CG treatment in human CCA cells (Supporting Fig. S2F). Enrichment of resistant cells expressing stem cell markers is known to occur in response to chemotherapy treatment in a variety of cancers.^22^, ^23^ Thus, we hypothesized that MIR1249 inhibition can increase chemo‐sensitivity by limiting the expansion of this resistant subpopulation. To verify our hypothesis, we assessed the expression of MIR1249 in human BTC spheroids generated from human cells freshly extracted from the stem cell niche of BTC samples, before and after selection for surface cell markers (Fig. 3A). MIR1249 expression was increased in BTC cells compared with noncancer biliary tract stem cells. CD133+ and CD13+ cells had increased expression of MIR1249 compared with CD133‐ and CD13 cells (Fig. 3A). CD133 has been consistently reported to be a marker of chemo‐resistant cancer cells, which are enriched after treatment,^22^, ^23^, ^24^, ^25^, ^26^, ^27^ and CD133+ BTC cells were shown to be tumorigenic and express features of cancer stem cells (CSCs).^9^, ^28^ Therefore, we speculated that MIR1249 could affect chemo‐resistance by inducing the expansion of CD133+ BTC cells. Indeed, an association between CD133 positivity and MIR1249 strong expression was observed in human BTC tissues (Supporting Fig. S2G). Increased MIR1249 expression was confirmed in CCLP‐1 CD133+ cells sorted from 2D cultures both by TaqMan assays and ISH (Fig. 3B). CD133+ cells gave rise to spheroids, indicating their self‐renewal properties (Fig. 3B‐C), and were more resistant to CG chemotherapy when cultured in 2D (Supporting Fig. S2H) or 3D (Fig. 3C‐D) in comparison to CD133‐ cells. Inhibition of MIR1249 in CD133+ CCLP‐1 cells increased sensitivity in comparison to CTRL miRNA inhibitor, whereas MIR1249‐enforced expression in CD133‐ cells reduced sensitivity to CG chemotherapy (Fig. 4A and Supporting Fig. S2I). To understand the potential of MIR1249 in controlling the expansion of CD133+ cells, we studied the fraction of CD133+ cells in the presence and absence of MIR1249 modulation. Enforced expression of MIR1249 in BTC cells expanded the proportion of the CD133+ subpopulation (Fig. 4B), which had increased expression of stem cell markers (Fig. 4C). Indeed, CSC markers were increased in CD133+ versus CD133‐ cells, and after transfection with MIR1249 mimic compared with mimic control (Fig. 4C). Conversely, inhibition of MIR1249 reduced the CG‐induced enrichment of CD133+ cells (Fig. 4D). To confirm the role of MIR1249 in the expansion of CD133+ cells, we generated a MIR1249 knockout (KO) CCLP‐1 cell line using CRISPR‐associated protein‐9 nuclease (CRISPR‐CAS9) technologies (Supporting Fig. S3A‐C). MIR1249KO cells were more sensitive to CG treatment with a concentration‐response effect (Fig. 4E) and showed impaired expansion of CD133+ cells (Supporting Fig. S3D and Fig. 4F), along with reduced expression of CSC markers (Supporting Fig. S3E) and lack of spheroid formation (Supporting Fig. S3F). Reintroduction of MIR1249 in MIR1249KO cells restored chemoresistance in CCLP‐1 cells (Fig. 4G and Supporting Fig. S3G).

### MIR1249 Drives Clonal Expansion of CD133+ Cells by Rewiring the Wnt Pathway Activation

**Figure 5:**
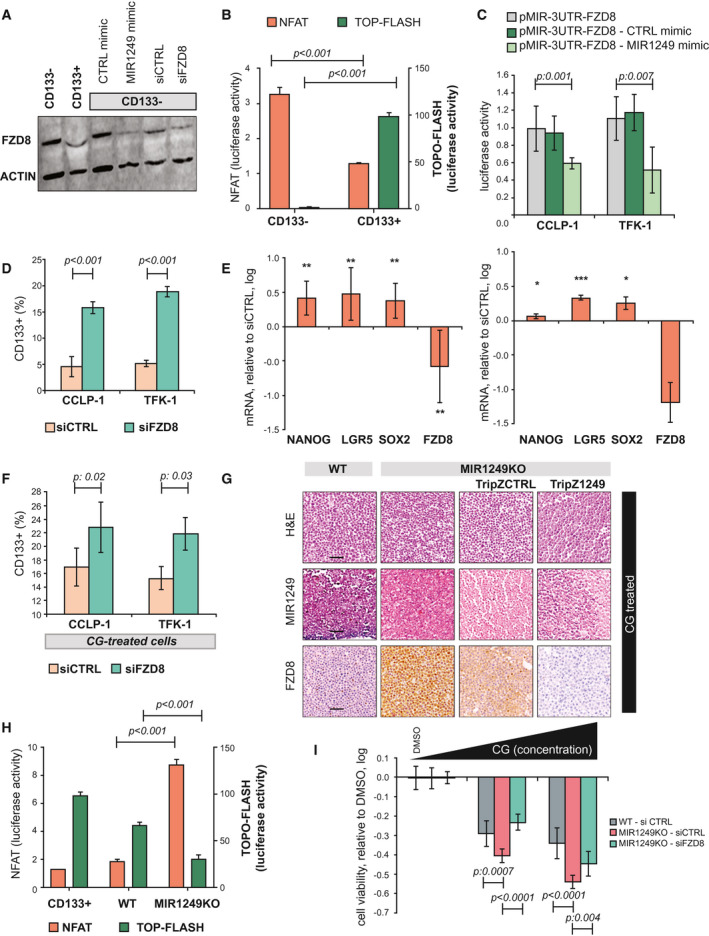
MIR1249 activates the Wnt pathway by acting on FZD8. (A) CCLP‐1 cells were sorted for CD133 expression and processed for western blot analysis (left side). CD133‐ cells were also transfected with the indicated probes for 48 hours before proceeding to western blot (right side). (B) CCLP‐1 cells were sorted and transfected with nuclear factor of activated T cells (NFAT) and TOP‐Flash vector for 48 hours before luciferase activity was recorded. Bars indicate the mean of six independent experiments ± SD. Two‐way analysis of variance < 0.05. (C) BTC cells were transfected with pMIR‐3UTR‐FZD8 vector ± MIR1249 mimic, and the luciferase activity was recorded. Bars indicate the mean of six independent experiments ± SD. (D) BTC cells were transfected for 48 hours, and positivity for CD133 was assessed by FACS. Bars represent the mean of three independent experiments ± SD. (E) CCLP‐1 (left) and TFK‐1 (right) cells were transfected with small interfering FZD8 or small interfering CTRL for 48 hours and collected for mRNA expression by TaqMan assay. Bars represent the log value of the ratio between siFZD8 and siCTRL. Bars indicate the mean of three independent experiments ± SD. *P < 0.05; **P < 0.01; ***P < 0.001. (F) CG‐treated BTC cells were transfected for 48 hours, and positivity for CD133 was assessed by FACS. Bars represent the mean of three independent experiments ± SD. (G) CCLP‐1 cells were infected with the indicated vectors and treated with CG chemotherapy before being fixed in formalin and embedded in paraffin for immunohistochemistry and ISH (see also Supporting Table S4). Scale bars: 100 μm. (H) Cells were transfected with NFAT or TOP‐Flash vectors, and luciferase activity was recorded after 48 hours. CD133+ cells were added as controls. Bars indicate the mean of three independent experiments ± SD. (I) CCLP‐1 cells were transfected for 48 hours and treated with DMSO or increasing doses of CG (up to 5 μM cisplatin and 50 nM gemcitabine). Bars represent the log value of the ratio between CG‐treated and DMSO‐treated cells. Bars indicate the mean of six independent experiments ± SD. Abbreviations: LGR5, leucine‐rich repeat‐containing G protein‐coupled receptor 5; siCTRL, small interfering CTRL; siFDZ8, small interfering FDZ8; SOX2, SRY (sex determining region Y)‐box 2.

To identify the mechanisms through which MIR1249 mediates chemo‐resistance, we characterized the gene‐expression profiles of chemotherapy‐treated CCLP‐1 cells after inhibition of MIR1249. Pathway analysis of the deregulated genes showed that MIR1249 inhibition induced changes in the same pathways that were deregulated by chemotherapy. We noticed an enrichment of deregulated genes in the Wnt pathway in both comparisons (chemotherapy vs. vehicle; MIR1249 inhibition vs. no inhibition), suggesting that MIR1249 inhibition may act on the Wnt pathway to restore chemotherapy sensitivity in BTC cells (Supporting Fig. S3H,I). In line with this hypothesis, Wnt deregulation was previously found to drive proliferation of chemo‐refractory CSCs.^29^, ^30^ PANTHER pathway analysis of the predicted targets of MIR1249 based on DIANA software showed an enrichment of Wnt signaling (fold enrichment 1.41; p:9.1E‐03). *In silico* analyses revealed, among others, frizzled class receptor 8 (FZD8) as a potential mRNA target of MIR1249 (Supporting Fig. S3L). Previous evidence has suggested that FZD8 can act as a negative regulator of the canonical Wnt pathway by activating the noncanonical Wnt/Ca^++^ signaling.^31^, ^32^, ^33^, ^34^ Thus, we hypothesized that MIR1249 mediates the expansion of CD133+ cells by acting on FZD8. Indeed, FZD8 was significantly reduced in CD133+ in comparison to CD133‐ cells and was associated with inactivation of the noncanonical Wnt and activation of the canonical Wnt pathway (Fig. 5A,B). FZD8 protein expression was reduced in CD133‐ cells transfected with MIR1249 mimic in comparison to CTRL mimic (Fig. 5A), and a luciferase reporter test confirmed a direct interaction between MIR1249 and the 3′ untranslated region (UTR) of FZD8 (Fig. 5C). In human BTC samples (cohort 3) there was a significant inverse relation between MIR1249 and FZD expression (Fisher exact test; *P* = 0.004; Supporting Fig. S3N). Inhibition of FZD8 recapitulated the phenotype induced by MIR1249 mimic (Fig. 5D‐F). MIR1249KO cells had increased activation of the noncanonical Wnt pathway (Fig. 5G‐H, Supporting Fig. S3M, and Supporting Table S4), and FZD8 inhibition in these cells partially increased resistance to CG (Fig. 5I).

### *In Vivo* Validation of the MIR1249‐Dependent Chemo‐resistance in Murine Tumors Bearing Disruption of MIR1249

**Figure 6:**
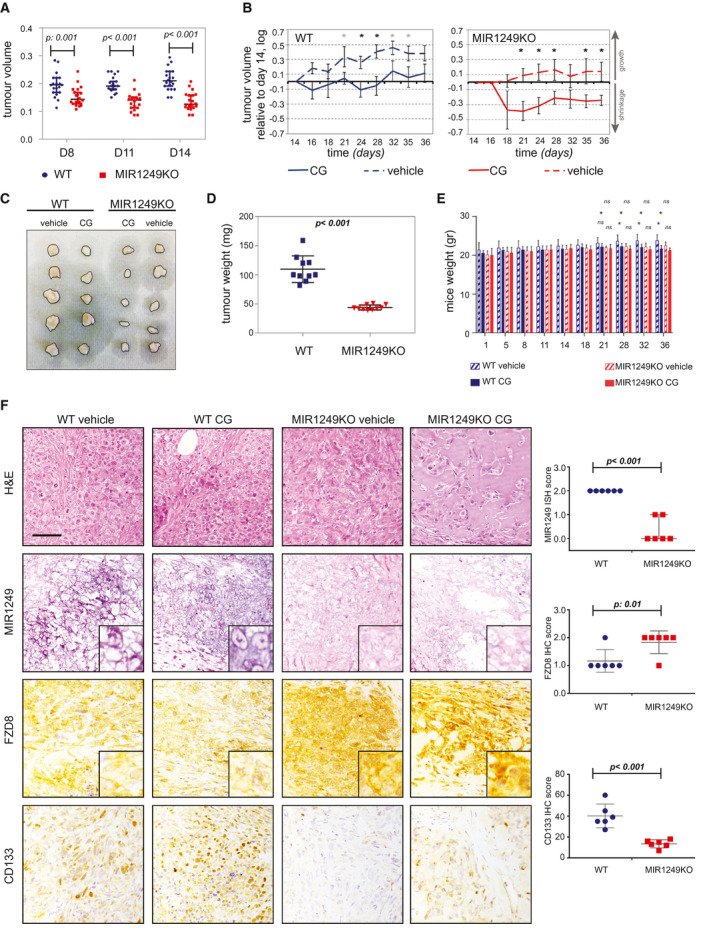
Lack of MIR1249 increases sensitivity to chemotherapy *in vivo*. (A) WT or MIR1249KO CCLP‐1 cells were injected subcutaneously in the flank of NSG (nonobese diabetic scid gamma) mice (n = 20 each) and monitored for growth by caliper (see also Supporting Fig. S4). (B) At day 14, mice were randomized to be treated with a weekly combination of intraperitoneal gemcitabine (150 mg/kg) and cisplatin (2 mg/kg) or vehicle alone for 3 weeks before being sacrificed. Data are presented normalized to baseline pretreatment tumor size (day 14). Black * indicates *P* value < 0.05; gray * indicates *P* value between 0.05 and 0.085 (see also Supporting Table S5). (C) Representative pictures of five explanted tumors per group. (D) Explanted tumors of CG‐treated mice were weighted before being stored for analyses. Error bars indicate the mean with SEM. (E) Mice were weighted periodically over the course of treatment. Bars represent the mean of 10 mice ± SD. *P < 0.05. (F) Representative pictures of MIR1249 ISH staining and immunohistochemistry staining for the indicated proteins performed on the explanted tumors, along with quantitative analysis. Scale bars: 100 μM. Three mice per group were analyzed (for a total of 12), and CG‐treated and vehicle‐treated tumors were grouped to assess the differences between KO and WT. Abbreviations: H&E, hematoxylin and eosin; ns, not significant.

MIR1249KO cells had reduced *in vivo* tumorigenicity, confirming the role of MIR1249 in driving the expansion of CSCs (Fig. 6A and Supporting Fig. S4A). Chemotherapy sensitivity was increased in mice bearing MIR1249KO tumor xenografts. Indeed, the weekly combination of CG could induce tumor shrinkage in MIR1249KO xenografts, while causing only tumor stabilization in wild‐type (WT) xenografts (Fig. 6B‐D and Supporting Table S5). The chemotherapy schedule was well‐tolerated with only minimal changes in weights for CG‐treated mice at the end of the treatment course. No differences in weights were observed between WT and MIR1249KO CG‐treated mice, suggesting that the drug exposure was comparable among the two groups (Fig. 6E). In addition, no differences in liver and kidney toxicity were observed between WT and MIR1249KO mice when treated with CG (Supporting Fig. SF4B‐C). To confirm the role of MIR1249 in driving the expansion of CD133+ cells through FZD8, we assessed the protein expression in explanted tumors from MIR1249KO versus WT xenograft (either treated with CG and vehicle) and observed that MIR1249KO tumors showed a lack of MIR1249 expression and reduced expression of CD133, with increase in FZD8 expression (Fig. 6F).

## Discussion

Therapeutic development for BTCs remains an unmet need. Chemotherapy does represent the main backbone for advanced biliary cancer (ABC) treatment, even though the response rate observed in patients with ABC does not exceed 25%. Strategies aimed at improving the efficacy of chemotherapy might prove beneficial to a large proportion of patients with ABC. The field of miRNA‐based therapeutics has recently expanded and entered the phase of clinical investigation.^10^, ^35^, ^36^ The ability of miRNAs to target multiple pathways is attractive, as it may prevent the onset of compensatory pathways.

Data from the MesomiR1 trial have shown the feasibility of a therapeutic approach based on miRNA replacement in human cancer patients.^37^ However, this approach holds two major limitations: (1) toxicity related to the immuno‐stimulatory effects of encapsulating delivery systems and (2) off‐target effects induced by a disproportionally high level of miRNA in the cellular system. Conversely, an approach based on the inhibition of miRNAs would reduce the risk of off‐target effects by affecting the physiological level of a miRNA rather than introducing a perturbation that affects cellular homeostasis. Recent technologies have enabled chemical modifications of anti‐miRNAs (i.e., the addition of LNA) that increase their stability,^38^ allowing the clinical investigation of this therapeutic strategy in cancer patients (NCT02580552). In these studies, we have identified MIR1249 as a miRNA that drives the emergence of chemo‐resistance by acting on the CD133+ cell population. Although we usually observe tumor stabilization with chemotherapy in patients with BTCs, our data suggest that the addition of MIR1249 inhibition to CG can increase tumor responses *in vivo*. It is known that partial responses are associated with prolonged life expectancy in patients with BTCs; therefore, we speculate that treatment with MIR1249 inhibitors might prove beneficial to affect the survival of patients with BTCs by preventing primary resistance and delaying the onset of secondary resistance. It has already been shown that the canonical Wnt/β‐catenin signaling mediates self‐renewal of stem cells, whereas noncanonical Wnt signaling pathways is involved in the maintenance of stem cells, cell plasticity, and inhibition of the canonical Wnt signaling cascade,^29^ supporting our data that MIR1249 can drive maintenance and expansion of CSCs through regulation of the noncanonical Wnt pathway. With regard to FZD8, so far, data in the literature are contradictory, with some reports showing the capacity of FZD8 to stimulate malignant transformation of cancer cells,^39^ and others showing its involvement in reducing tumor initiating capacity.^32^ In line with our data that showed low expression of FZD8 in CSCs, analysis of the TCGA data sets showed unfavorable prognosis in the cases of pancreatic cancers with low FZD8 expression. Nonetheless, attempts to therapeutically inhibit FZD8 have failed so far due to lack of therapeutic index, suggesting that FZD8 may not be involved in promoting cancer growth.

Finally, in this study the combination of CG was given in a weekly schedule to BTC mouse models in an attempt to better mimic the schedule used in the ABC‐02 trial, which licensed the combination CG for standard clinical practice. We noticed good tolerance of this schedule and suggest that this regimen be used in future *in vivo* BTC modeling, to increase the clinical relevance of preclinical findings.

In conclusion, we have provided evidence for a potential target to be considered in future therapeutic development. Our data suggest that MIR1249 is involved in the chemo‐resistance in all different subtypes of CCA; therefore, we suggest testing the MIR1249 inhibitor in a trial including ABCs. In addition, it may be speculated that this mechanism may be shared with other cancer types as well, which may warrant investigation in other solid tumors.

## Author Contributions

C.B. was responsible for the study concept and design. P.C., S.H., M.F., A.L., V.G., C.V., M.G., F.T., I.S.H., R.T.P., M.S., V.G., S.V., G.V., and J.C.H. were responsible for the data acquisition. P.C. and C.B. were responsible for the analysis and interpretation of data. P.C. was responsible for drafting of the manuscript. C.B. was responsible for critical revision of the manuscript for important intellectual content. L.C. and C.B. were responsible for the statistical analysis. V.C., A.S., D.C., D.A., N.V., L.B., R.G., S.J.F., M.R., U.C., R.B., E.S., V.M., D.C., L.R., A.S., P.C., and V.K. were responsible for material support. C.B., N.V., and P.W. were responsible for obtaining funding.

## References

[1] Khan SA , Emadossadaty S , Ladep NG , Thomas HC , Elliott P , Taylor‐Robinson SD , et al. Rising trends in cholangiocarcinoma: is the ICD classification system misleading us? J Hepatol 2012;56:848‐85 4. 22173164 10.1016/j.jhep.2011.11.015

[2] Bridgewater J , Galle PR , Khan SA , Llovet JM , Park JW , Patel T , et al. Guidelines for the diagnosis and management of intrahepatic cholangiocarcinoma. J Hepatol 2014;60:1268‐1289. 24681130 10.1016/j.jhep.2014.01.021

[3] Marcano‐Bonilla L , Mohamed EA , Mounajjed T , Roberts LR . Biliary tract cancers: epidemiology, molecular pathogenesis and genetic risk associations. Chin Clin Oncol 2016;5:61. 27829275 10.21037/cco.2016.10.09

[4] Saha SK , Zhu AX , Fuchs CS , Brooks GA . Forty‐year trends in cholangiocarcinoma incidence in the U.S.: intrahepatic disease on the rise. Oncologist 2016;21:594‐599. 27000463 10.1634/theoncologist.2015-0446 PMC4861366

[5] Valle J , Wasan H , Palmer DH , Cunningham D , Anthoney A , Maraveyas A , et al. Cisplatin plus gemcitabine versus gemcitabine for biliary tract cancer. N Engl J Med 2010;362:1273‐1281. 20375404 10.1056/NEJMoa0908721

[6] Bridgewater J , Lopes A , Palmer D , Cunningham D , Anthoney A , Maraveyas A , et al. Quality of life, long‐term survivors and long‐term outcome from the ABC‐02 study. Br J Cancer 2016;114:965‐971. 27115567 10.1038/bjc.2016.64 PMC4984909

[7] Marin JJ , Briz O , Rodriguez‐Macias G , Diez‐Martin JL , Macias RI . Role of drug transport and metabolism in the chemoresistance of acute myeloid leukemia. Blood Rev 2016;30:55‐64. 26321049 10.1016/j.blre.2015.08.001

[8] Ishiwata T . Cancer stem cells and epithelial‐mesenchymal transition: Novel therapeutic targets for cancer. Pathol Int 2016;66:601‐608. 27510923 10.1111/pin.12447

[9] Huang L , Cai J , Guo H , Gu J , Tong Y , Qiu B , et al. ID3 promotes stem cell features and predicts chemotherapeutic response of intrahepatic cholangiocarcinoma. Hepatology 2019;69:1995‐2012. 30520117 10.1002/hep.30404

[10] Salati M , Braconi C . Noncoding RNA in cholangiocarcinoma. Semin Liver Dis 2019;39:13‐25. 30536290 10.1055/s-0038-1676097

[11] Meng F , Henson R , Wehbe‐Janek H , Ghoshal K , Jacob ST , Patel T . MicroRNA‐21 regulates expression of the PTEN tumor suppressor gene in human hepatocellular cancer. Gastroenterology 2007;133:647‐658. 17681183 10.1053/j.gastro.2007.05.022 PMC4285346

[12] Selaru FM , Olaru AV , Kan T , David S , Cheng Y , Mori Y , et al. MicroRNA‐21 is overexpressed in human cholangiocarcinoma and regulates programmed cell death 4 and tissue inhibitor of metalloproteinase 3. Hepatology 2009;49:1595‐1601. 19296468 10.1002/hep.22838 PMC3124086

[13] Braconi C , Huang N , Patel T . MicroRNA‐dependent regulation of DNA methyltransferase‐1 and tumor suppressor gene expression by interleukin‐6 in human malignant cholangiocytes. Hepatology 2010;51:881‐890. 20146264 10.1002/hep.23381 PMC3902044

[14] Braconi C , Valeri N , Gasparini P , Huang N , Taccioli C , Nuovo G , et al. Hepatitis C virus proteins modulate microRNA expression and chemosensitivity in malignant hepatocytes. Clin Cancer Res 2010;16:957‐966. 20103677 10.1158/1078-0432.CCR-09-2123 PMC2818698

[15] Braconi C , Valeri N , Kogure T , Gasparini P , Huang N , Nuovo GJ , et al. Expression and functional role of a transcribed noncoding RNA with an ultraconserved element in hepatocellular carcinoma. Proc Natl Acad Sci U S A 2011;108:786‐791. 21187392 10.1073/pnas.1011098108 PMC3021052

[16] Carotenuto P , Fassan M , Pandolfo R , Lampis A , Vicentini C , Cascione L , et al. Wnt signalling modulates transcribed‐ultraconserved regions in hepatobiliary cancers. Gut 2017;66:1268‐1277. 27618837 10.1136/gutjnl-2016-312278 PMC5530482

[17] Braconi C , Patel T . Cholangiocarcinoma: new insights into disease pathogenesis and biology. Infect Dis Clin North Am 2010;24:871‐884, vii. 20937455 10.1016/j.idc.2010.07.006 PMC2954129

[18] Lampis A , Carotenuto P , Vlachogiannis G , Cascione L , Hedayat S , Burke R , et al. MIR21 drives resistance to heat shock protein 90 inhibition in cholangiocarcinoma. Gastroenterology 2018;154:1066‐1079.e5. 29113809 10.1053/j.gastro.2017.10.043 PMC5863695

[19] Braconi C , Roessler S , Kruk B , Lammert F , Krawczyk M , Andersen JB . Molecular perturbations in cholangiocarcinoma: Is it time for precision medicine? Liver Int 2019;39(Suppl. 1):32‐42. 30829432 10.1111/liv.14085

[20] Meng F , Henson R , Wehbe‐Janek H , Smith H , Ueno Y , Patel T . The MicroRNA let‐7a modulates interleukin‐6‐dependent STAT‐3 survival signaling in malignant human cholangiocytes. J Biol Chem 2007;282:8256‐8264. 17220301 10.1074/jbc.M607712200

[21] Farshidfar F , Zheng S , Gingras MC , Newton Y , Shih J , Robertson AG , et al. Integrative genomic analysis of cholangiocarcinoma identifies distinct IDH‐mutant molecular profiles. Cell Rep 2017;19:2878‐2880. 28658632 10.1016/j.celrep.2017.06.008 PMC6141445

[22] Steg AD , Bevis KS , Katre AA , Ziebarth A , Dobbin ZC , Alvarez RD , et al. Stem cell pathways contribute to clinical chemoresistance in ovarian cancer. Clin Cancer Res 2012;18:869‐881. 22142828 10.1158/1078-0432.CCR-11-2188 PMC3271164

[23] Auffinger B , Tobias AL , Han Y , Lee G , Guo D , Dey M , et al. Conversion of differentiated cancer cells into cancer stem‐like cells in a glioblastoma model after primary chemotherapy. Cell Death Differ 2014;21:1119‐1131. 24608791 10.1038/cdd.2014.31 PMC4207480

[24] Suetsugu A , Nagaki M , Aoki H , Motohashi T , Kunisada T , Moriwaki H . Characterization of CD133+ hepatocellular carcinoma cells as cancer stem/progenitor cells. Biochem Biophys Res Commun 2006;351:820‐824. 17097610 10.1016/j.bbrc.2006.10.128

[25] Suetsugu A , Osawa Y , Nagaki M , Moriwaki H , Saji S , Bouvet M , et al. Simultaneous color‐coded imaging to distinguish cancer “stem‐like” and non‐stem cells in the same tumor. J Cell Biochem 2010;111:1035‐1041. 20672309 10.1002/jcb.22792

[26] Cortes‐Dericks L , Carboni GL , Schmid RA , Karoubi G . Putative cancer stem cells in malignant pleural mesothelioma show resistance to cisplatin and pemetrexed. Int J Oncol 2010;37:437‐444. 20596671 10.3892/ijo_00000692

[27] Kelly SE , Di Benedetto A , Greco A , Howard CM , Sollars VE , Primerano DA , et al. Rapid selection and proliferation of CD133+ cells from cancer cell lines: chemotherapeutic implications. PLoS ONE 2010;5:e10035. 20386701 10.1371/journal.pone.0010035 PMC2851647

[28] Cardinale V , Renzi A , Carpino G , Torrice A , Bragazzi MC , Giuliante F , et al. Profiles of cancer stem cell subpopulations in cholangiocarcinomas. Am J Pathol 2015;185:1724‐1739. 25892683 10.1016/j.ajpath.2015.02.010 PMC4450332

[29] Katoh M . Canonical and non‐canonical WNT signaling in cancer stem cells and their niches: cellular heterogeneity, omics reprogramming, targeted therapy and tumor plasticity (Review). Int J Oncol 2017;51:1357‐1369. 29048660 10.3892/ijo.2017.4129 PMC5642388

[30] Mohammed MK , Shao C , Wang J , Wei Q , Wang X , Collier Z , et al. Wnt/beta‐catenin signaling plays an ever‐expanding role in stem cell self‐renewal, tumorigenesis and cancer chemoresistance. Genes Dis 2016;3:11‐40. 27077077 10.1016/j.gendis.2015.12.004 PMC4827448

[31] Semenov MV , Habas R , Macdonald BT , He X . SnapShot: noncanonical Wnt signaling pathways. Cell 2007;131:1378. 18160045 10.1016/j.cell.2007.12.011

[32] Wang MT , Holderfield M , Galeas J , Delrosario R , To MD , Balmain A , et al. K‐Ras promotes tumorigenicity through suppression of non‐canonical Wnt signaling. Cell 2015;163:1237‐1251. 26590425 10.1016/j.cell.2015.10.041

[33] Sugimura R , Li L . Noncanonical Wnt signaling in vertebrate development, stem cells, and diseases. Birth Defects Res C Embryo Today 2010;90:243‐256. 21181886 10.1002/bdrc.20195

[34] Masoumi KC , Daams R , Sime W , Siino V , Ke H , Levander F , et al. NLK‐mediated phosphorylation of HDAC1 negatively regulates Wnt signaling. Mol Biol Cell 2017;28:346‐355. 27903773 10.1091/mbc.E16-07-0547 PMC5231902

[35] Rupaimoole R , Slack FJ . MicroRNA therapeutics: towards a new era for the management of cancer and other diseases. Nat Rev Drug Discov 2017;16:203‐222. 28209991 10.1038/nrd.2016.246

[36] Constantinescu CA , Fuior EV , Rebleanu D , Deleanu M , Simion V , Voicu G , et al. Targeted transfection using PEGylated cationic liposomes directed towards P‐selectin increases siRNA delivery into activated endothelial cells. Pharmaceutics 2019;11:47. 10.3390/pharmaceutics11010047 PMC6359248 30669699

[37] van Zandwijk N , Pavlakis N , Kao SC , Linton A , Boyer MJ , Clarke S , et al. Safety and activity of microRNA‐loaded minicells in patients with recurrent malignant pleural mesothelioma: a first‐in‐man, phase 1, open‐label, dose‐escalation study. Lancet Oncol 2017;18:1386‐1396. 28870611 10.1016/S1470-2045(17)30621-6

[38] Cheng CJ , Bahal R , Babar IA , Pincus Z , Barrera F , Liu C , et al. MicroRNA silencing for cancer therapy targeted to the tumour microenvironment. Nature 2015;518:107‐110. 25409146 10.1038/nature13905 PMC4367962

[39] Yin S , Xu L , Bonfil RD , Banerjee S , Sarkar FH , Sethi S , et al. Tumor‐initiating cells and FZD8 play a major role in drug resistance in triple‐negative breast cancer. Mol Cancer Ther 2013;12:491‐498. 23445611 10.1158/1535-7163.MCT-12-1090 PMC3624033

