# Supplementary material for: Modulation of Biliary Cancer Chemo‐Resistance Through MicroRNA‐Mediated Rewiring of the Expansion of CD133+ Cells
Source: Hepatology. 2020 Sep 10;72(3):982–96. doi: 10.1002/hep.31094 (PMC7590111; doi:10.1002/hep.31094)
Supplement: Supplementary file 1 [file HEP-72-982-s001.pdf]

A

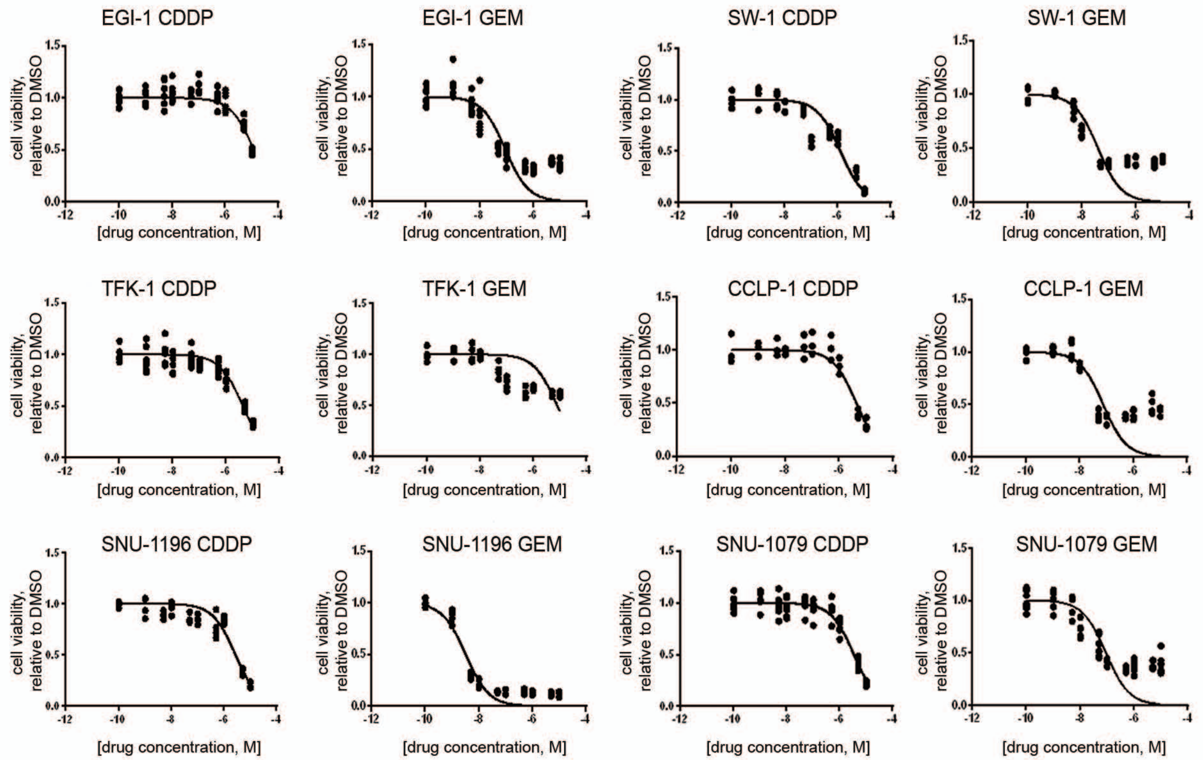

B

Cell line	Origin	CDDP, GI50 (μM)	GEM, GI50 (nM)
EGI-1	dCCA	13	93
TFK-1	dCCA	4.4	8000
SNU-1196	pCCA	2.8	3.4
SW1	iCCA	0.7	42
CCLP-1	iCCA	4.4	77
SNU-1079	iCCA	4.5	95

C

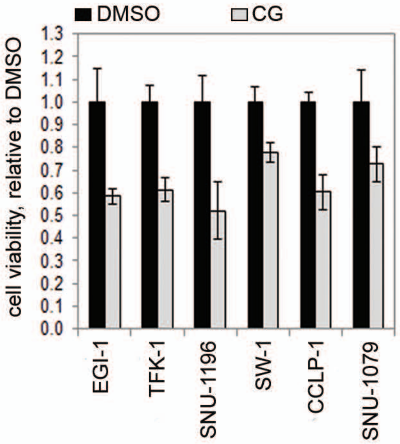

	CDDP, μM	GEM, nM
EGI-1	10	50
TFK-1	3	8000
SNU-1196	2	2
SW-1	0.5	10
CCLP-1	3	20
SNU-1079	3	10

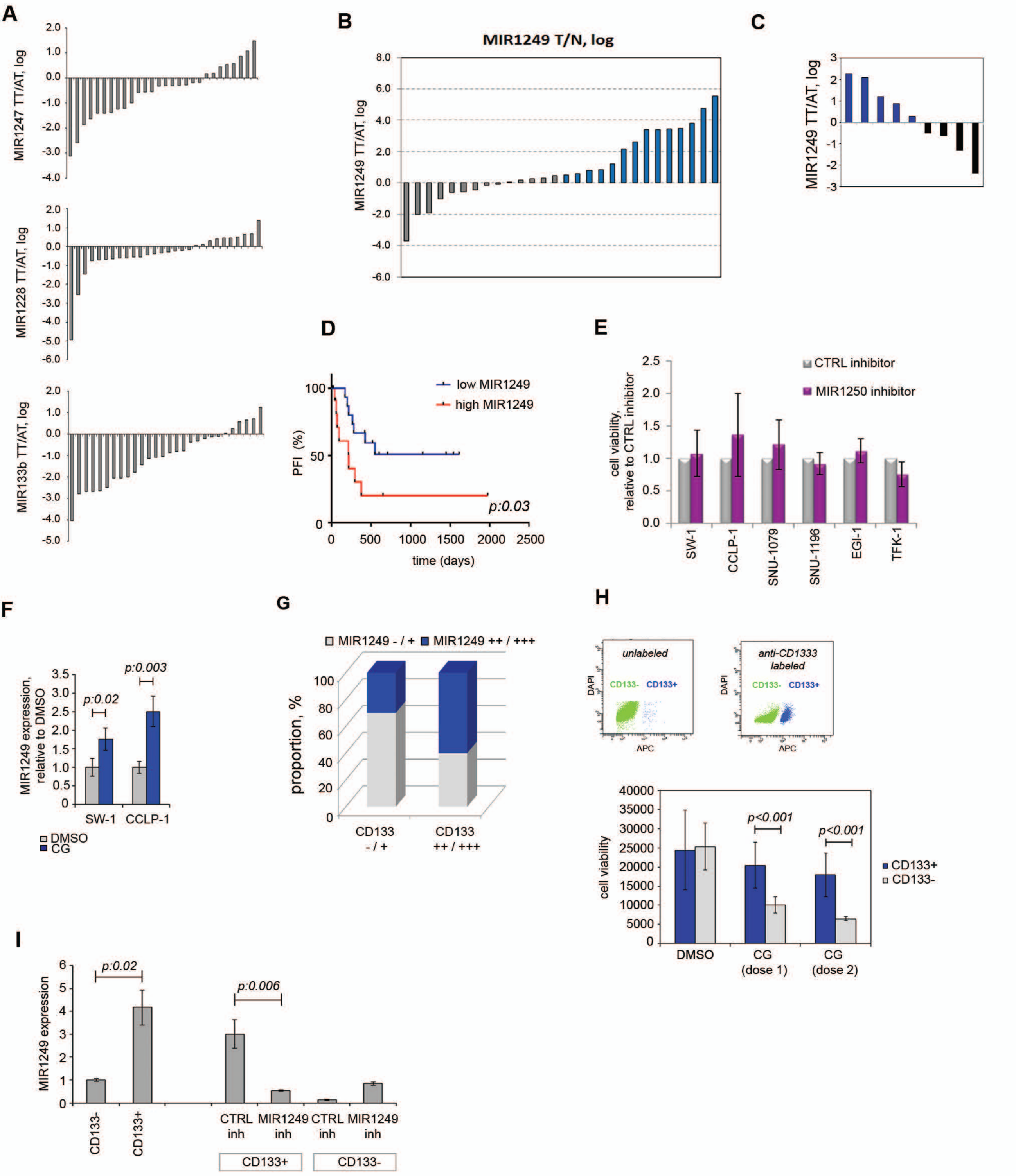
A
MIR1247 TT/AT, log
MIR1228 TT/AT, log
MIR133b TT/AT, log
B
MIR1249 T/N, log
MIR1249 TT/AT, log
C
MIR1249 TT/AT, log
D
low MIR1249
high MIR1249
PFI (%)
time (days)
p:0.03
E
CTRL inhibitor
MIR1250 inhibitor
cell viability, relative to CTRL inhibitor
SW-1
CCLP-1
SNU-1079
SNU-1196
EGI-1
TFK-1
F
MIR1249 expression, relative to DMSO
p:0.02
p:0.003
SW-1
CCLP-1
DMSO
CG
G
MIR1249 - / +
MIR1249 ++ / +++
proportion, %
CD133 - / +
CD133 ++ / +++
H
unlabeled
anti-CD1333 labeled
CD133-
CD133+
DAPI
APC
cell viability
p<0.001
CD133+
CD133-
DMSO
CG (dose 1)
CG (dose 2)
I
MIR1249 expression
p:0.02
p:0.006
CD133-
CD133+
CTRL inh
MIR1249 inh
CD133+
CD133-

SUPPL FIGURE 3

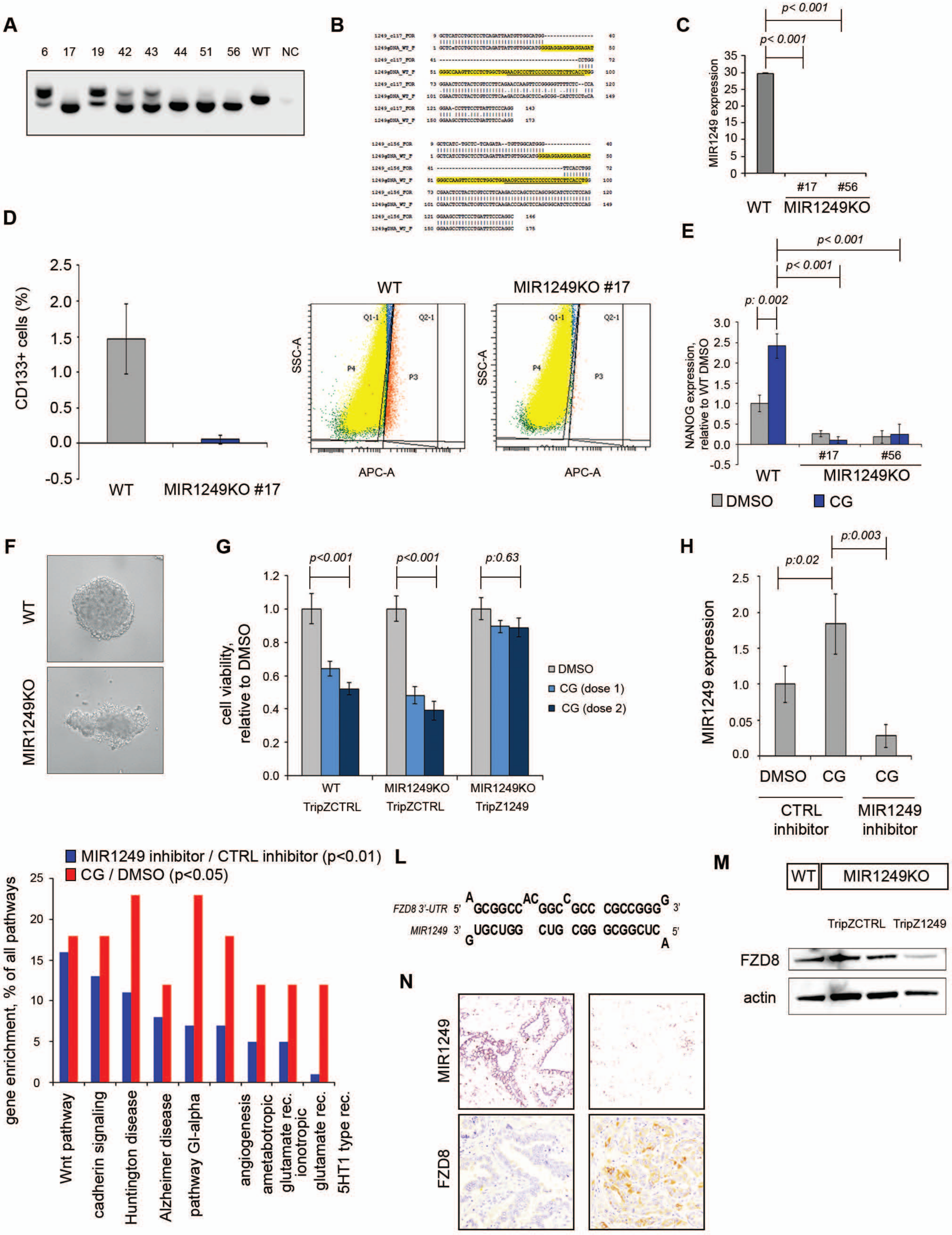

SUPPL FIGURE 4

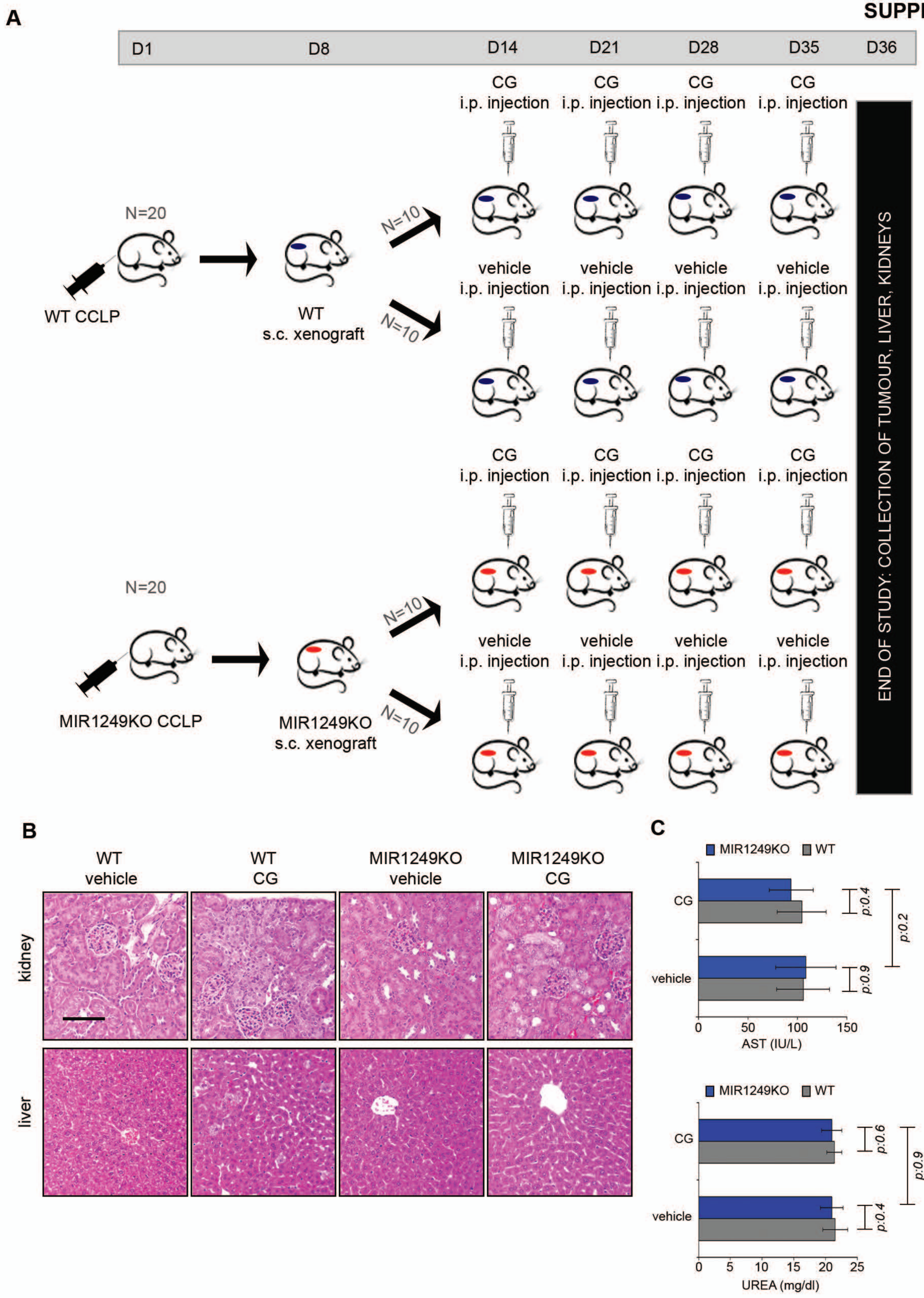

Modulation of biliary cancer chemo-resistance through microRNA-mediated rewiring of the expansion of CD133+ cells

Short title: MIR1249 as mediator of chemo-resistance in cholangiocarcinoma (CCA)

Pietro Carotenuto[1-2], Somaieh Hedayat[3], Matteo Fassan[4], Vincenzo Cardinale[5], Andrea Lampis[3], Vincenza Guzzardo[4], Caterina Vicentini[6], Aldo Scarpa[6], Luciano Cascione[7], Daniele Costantini[5], Guido Carpino[8], Domenico Alvaro[9], Michele Ghidini[1-10], Francesco Trevisani[3], Robert Te Poele[1], Massimiliano Salati[1], Sofia Ventura[1], Georgios Vlachogiannis[3], Jens C Hahne[3], Luke Boulter[11], Stuart J Forbes[12], Rachel Guest[12], Umberto Cillo[4], Ian Said-Huntingford[3], Ruwaida Begum[13], Elizabeth Smyth[13], Vasiliki Michalarea[13], David Cunningham[13], Lorenza Rimassa[10-14], Armando Santoro[10-14], Massimo Roncalli[15], Vladimir Kirnkin[1], Paul Clarke[1], Paul Workman[1], Nicola Valeri[3,13], Chiara Braconi[1,13,16*]

[1] Division of Cancer Therapeutics, Institute of Cancer Research, SM2 5NG, London, UK;

[2] Telethon Institute of Genetics and Medicine, Via Campi Flegrei 34, 80078 Pozzuoli (NA), Italy

[3] Division of Molecular Pathology, Institute of Cancer Research, SM2 5NG, London, UK;

[4] Department of Medicine, Surgical Pathology Unit, University of Padua, 35139, Padua, IT;

[5] Department of Medico-Surgical Sciences and Biotechnologies, Sapienza University of Rome, Latina, 04100, IT

[6] Department of Pathology, University of Verona, 37126, Verona, IT;

[7] Bioinformatics Core Unit, Institute of Oncology Research, 6500, Bellinzona, CH;

[8] Department of Movement, Human and Health Sciences, University of Rome Foro Italico, Rome, 00135, IT

[9] Department of Translational and Precision Medicine, Sapienza University, 00185, Rome, IT;

[10] Medical Oncology and Hematology Unit, Humanitas Cancer Center, Humanitas Clinical and Research Center-IRCCS, 20089, Rozzano, IT

[11] MRC Human Genetics Unit, Institute of Genetics and Molecular Medicine, EH4 2XU. Edinburgh, UK;

[12] Centre for Regenerative Medicine, University of Edinburgh, EH16 4UU. Edinburgh, UK;

[13] The Royal Marsden NHS Trust Surrey and London, SM2 5TP, Sutton, UK;

[14] Department of Biomedical Sciences, Humanitas University, 20089, Rozzano, IT

[15] Department of Pathology, Humanitas Research Hospital & Hunimed University, Rozzano (Milan) Italy

[16] The Institute of Cancer Sciences, University of Glasgow, G61 1QH, Glasgow, UK

*Corresponding and lead author: Chiara Braconi, The Institute of Cancer Science, University of Glasgow, Glasgow, UK, G61 1QH. Phone: +44(0) 141 330 3278. Fax: 0141 942 6521 Email: chiara.braconi@glasgow.ac.uk

SUPPLEMENTARY INFORMATION

Methods

Cell lines. Intrahepatic (SW-1, SNU-1079, CCLP-1), and extrahepatic (SNU-1196, TFK-1, EGI-1) CCA cell lines were purchased from the Leibniz Institute DSMZ-German Collection of Microorganisms and Cell Culture (Braunschweig, Germany), the Korean Cell Line Bank (Seoul, Korea) or were kindly provided by Prof. Stuart Forbes (University of Edinburgh). Cells were cultured in Dulbecco's modified Eagle medium with 10 % foetal bovine serum, at 37°C with 5% carbon dioxide. Authentication of cell lines was achieved by Short tandem repeat (Surrey Diagnostics, Cranleigh, UK).

Cell viability. Cell viability was measured by CellTiter-Blue® Assay (Promega, Madison, WI, USA) and the GI_{50} derived using Prism Software (Graphpad, La Jolla, USA).

Validation experiments. The same protocol used for the HTS was used for the validation experiments. However, miRvana inhibitors probes were used (Life Technologies, Paisley, UK), and cells were treated with either a combination of chemotherapy (CG) or with DMSO.

Real time PCR. Reverse transcription was performed with Taqman microRNA reverse transcription kit, and miRNA expression assessed by qPCR with TaqMan® MicroRNA Assays (Life Technologies, Paisley, UK). miRNA expression was normalized to that of RNU48 (TaqMan® MicroRNA Assays, Life Technologies, Paisley, UK). For CSC Markers, the reverse transcription was performed using High Capacity cDNA Kit (Life Technologies, Paisley, UK), and NANOG, LGR5 and SOX2 mRNA expression were assessed using the following Taqman Gene Expression assays Hs01053049_s1 (SOX2), Hs00969422_m1 (LGR5),

Hs02387400_g1 (NANOG) and TaqMan™ Fast Advanced Master Mix following the manufacturer instructions (Life Technologies, Paisley, UK).

In Situ RNA hybridization. A locked nucleic acid (LNA) probe with complementarity to MIR1249 was labelled with 5′-digoxigenin and synthesized by Exiqon (Vedbaek Denmark). Tissue sections were digested with ISH protease 1 (Ventana Medical Systems Roche, Basel, Switzerland) and *in situ* hybridization performed as described [1]. Negative controls included omission of the probe and the use of a scrambled LNA probe. Each sample was classified according to a 4-tiered scoring system based on the intensity of MIR1249 expression as follows: 0: indicates no stain or stain in less than 10% of tumour cells; 1+: faint/weak cytoplasm/nuclear stain in 10% or more of tumour cells; 2+: moderate cytoplasm/nuclear stain in 10% or more of tumour cells; and 3+: strong cytoplasm/nuclear stain in 10% or more of tumour cells. In case of ISH on cell pellet, the latter was fixed in formalin and embedded in paraffin before applying the protocol above described.

Immunohistochemistry. Immunohistochemical stains were automatically performed in 3-4 μm sections using the Bond Polymer Refine Detection kit in the BOND-MAX system (Leica Biosystems, Newcastle upon Tyne, UK), according to the manufacturer's specifications. Appropriate positive and negative controls were run concurrently. The following antibodies were used: CD133 (HPA004922, Atlas Antibodie, Bromma, Sweden) FZD8 (HPA045025, Atlas Antibodies AB, Bromma, Sweden). Protein expression was classified according to a 4-tiered scoring system based on the intensity of protein expression as follows: 0: indicates no stain or stain in less than 10% of tumour cells; 1+: faint/weak cytoplasm/nuclear stain in 10% or more of tumour cells; 2+: moderate cytoplasm/nuclear stain in 10% or more of tumour cells; and 3+: strong cytoplasm/nuclear stain in 10% or more of tumour cells.

FACS for sorting and analysis of CD133+cells. Flow cytometry analysis was carried out on a fluorescence-activated cell sorting (FACS) caliber (BD FACSAriaIIμ and BD FACSDiva Software (Becton Dickinson, Swindon, UK). Cholangiocarcinoma cells (1×10^6) were collected with 0.05% trypsin–EDTA solution, washed, and diluted to 1 million cells per ml in PBS/2%FCS, and stained with human CD133/1 (1:10, APC conjugated, AC133, PE,130-080-

801; Miltenyi Biotec, Woking, UK) and 1 µg/mL propidium iodide (PI) to exclude dead cells during sorting. Matched isotype antibodies were applied in parallel as controls. For CD133+ cell counting a total of 30,000 viable cells were counted for each sample. All samples were analyzed in triplicate.

***Transfection*.** Cells were reverse-transfected in 96-well plates using HiPerFect Transfection Reagent Qiagen, Hilden, Germany). For transient inhibition of miRNAs miRvana inhibitors (Ambion-Life Technologies, Paisley, UK) or miRNA mimic (Ambion-Life Technologies, Paisley, UK) were used at 50 nM concentrations. siFZD8 was obtained from GE Healthcare Dharmacon, Little Chalfont, UK (ON-TARGETplus Human SiRNA).

Stable overexpression of MIR1249. MIR1249KO CCLP-1 cells were infected with TRIPZ Inducible Lentiviral shRNA (GE Healthcare Dharmacon, Little Chalfont, UK) in which pre-MIR1249 sequence (or a scramble control) was cloned using PCR amplification of target region and digestion with Cla I and Mlu I restriction enzymes (New England Biolabs, Ipswich, Massachusetts, USA). The lentiviral infection of TRIPZ1249 and TRIPZCTRL was performed using Trans-Lentiviral shRNA Packaging Kit, according to manufacturer's instructions. The precursor MIR1249 sequence Packaging of viral particles and target cell lines infections were performed using HEK293T. Overexpression of MIR1249 was confirmed by Taqman assay.

3D-Spheroid formation. CD133+ and CD133- cells were isolated through FACS. In case of transfections, cells were subjected to reverse transfection using RNAiMAX (Invitrogen, Carlsbad, CA, USA) in 96 well plates or 6 well plates as per the manufacturer's instruction. After 24 hours cells were plated in UltraLow Attachment (ULA) round bottom 96 well plates (Corning, Corning, NY, USA) and 3D-spheroid formation analysed with Celigo S (Nexcelom, Manchester, UK) as previously described [2].

***Western Blotting*.** Cells were collected and protein extracted. Immunoblot analysis was performed using the following antibodies: Mouse Monoclonal Anti-Actin (MP Biomedical, Santa Ana, CA, USA), Anti-Frizzled 8 antibody (ab40012, Abcam, Cambridge, UK).

Luciferase assays. *For miRNA-target assay,* cells were transfected with 1 µg pMirTarget-3'-UTR-FZD8 or pMirTarget 3'-UTR Assay Vector CTRL (Origene, Rockville, USA) with

HiPerFect Transfection Reagent (Qiagen, Hilden, Germany). For WNT-pathway transcriptional activation assay, cells were plated to form 60 to 80% confluent cultures in 96-well dishes in triplicates and transfected with pGL4.30[luc2P/NFAT-RE/Hygro] (NFAT) and pGL4.49[luc2P/TCF-LEF RE/Hygro] (TOP-FLASH) luciferase reporter plasmids (Promega, Madison, WI, USA) with Lipofectamine 2000 (Thermofisher, Waltham, MA, USA). NFAT measures the activation of the Ca++ dependent non-canonical Wnt pathway, while the TOP-FLASH assess the activation of the canonical b-catenin. Dependent Wnt pathway. HEPG2 cells were used as a positive CTRL of WNT-pathway transcriptional activation. The luciferase activity measured after 48h using the Dual-Glo® Luciferase Assay System (Promega, Madison, WI, USA) according to the manufacturer's protocol in a multiwell plate luminometer (Perkin-Elmer, Seer Green, Beaconsfield, UK). Luciferase activity was normalized to that of renilla activity for each transfected well.

Microarray analysis and GO analysis. Total RNA has been isolated using TRIZOL reagent (Life technologies, Paisley, UK). Then, each sample was amplified and transcribed into fluorescent cRNA using a random priming method. The labelled cRNAs were hybridized and scanned by the Agilent Scanner G2505C (Agilent Technologies, Santa Clara, California, USA). Agilent Feature Extraction software (V.11.0.1.1) was used to analyze the acquired array images. Sample-specific background was calculated by adding the average of all the negative controls to their standard deviation multiplied by two and then subtracting this value from the raw values for each gene. Biological normalization was performed to correct for differences in sample abundances. Each sample was normalized according to the quantile normalization method, and the data were log transformed (base 2) for analysis. The linear model for microarray (LIMMA) package in R was used on normalized counts to calculate statistical significances of pairwise comparisons. P-value was adjusted for multiple comparisons by Benjamini-Hochberg methods. Significance was accepted when p was less than *0.0*5. GO pathway enrichment analysis analyses were performed using PANTHER database Version 14.0 (released 2018-12-03) available under the permission of Thomas lab at the University of Southern California.

Spheroid generation from human biliary stem cell niche of BTC patients. Human BTC samples were subjected to mechanical and enzymatic dissociation by mincing them in small volume DMEM high glucose/DMEM:F12 mixture (Gibco/BRL, Life Technologies, Italia srl., Milan, Italy) supplemented with 1.8 x 10^{-4} mol/L adenine, 5 µg/ml insulin, 5 µg/ml transferrin, 2 x 10^{-9} mol/L triiodothyronine, 1.7 x 10^{-6} mol/L hydrocortisone, 1.0 x 10^{-6} human epidermal growth factor, 5.5 x 10^{-6} epinephrine (Sigma-Aldrich, Milan, Italy), 10% fetal bovine serum (Gibco/BRL, Life Technologies, Milan, Italy), 100 U/ml penicillin, and 100 µg/ml streptomycin. The cells were re-suspended in growth medium and placed in to 6-well dish at 37°C in a humidified atmosphere of 5% CO2 in air. Primary BTC cell cultures were characterized at cell culture passages 2-3 and 20-30 by using the following antibodies: PE-mouse anti-human CD13 (BD Pharmigen, Milan, Italy), CD90-FITC human, CD133-APC human, EpCAM-FITC human (Miltenyi Biotec, Milan, Italy) (Origene, Unimed Scientifica, Rome, Italy). The fluorescence threshold between negative and positive cells was set on the basis of the reactivity of appropriate non-specific fluorochrome-conjugated isotypic controls. At least 5 X 10^5 cells were analyzed using a FACSDiva software (BD). Cells were labelled with CD133 (AC133), CD326 (EPCAM), CD90 MicroBeads, and CD13 (BD Pharmigen, Milan, Italy), and sorted using the Miltenyi Biotec Cell Isolation Kit, according to manufacturer's instruction. Two-thousands sorted cells (CD133+/CD133-) were cultured in a serum-free medium DMEM with high glucose/DMEM:F12 mixture (1:1) (Gibco/BRL, Life Technologies, Italia srl., Milan, Italy) supplemented with 20ng/ml^{-1} EGF, 10ng/ml^{-1} FGF-2 and 1x B27 (Gibco/BRL, Life Technologies, Italia srl., Milan, Italy) in ultra-low attachment plates (Corning, Lowell, MA). After seven days, spheres were visualized, counted and sized using light microscopy.

CCLP-1 BTC Xenograft model. BTC xenograft tumours (WT N:20; MIR1249KO N:20) were established subcutaneously in 6-7 weeks female NOD-scid IL2Rgnull mice (Charles River Laboratories, Wilmington, MA, USA). A total of 7x10E+6 CCLP-1 cells in 100% Matrigel (BD Biosciences, Wokingham, UK) were injected in a single flank. Two weeks post inoculation, mice were randomly grouped for initiation of treatment with weekly intraperitoneal (i.p.) administration of gemcitabine at 150mg/kg (0.9% sodium chloride) and cisplatin at 2mg/kg

(0.9% sodium chloride) or vehicle alone for three weeks. Tumour volumes and mouse body weights were determined at regular intervals. Tumour volume was determined using the following formula: =4.19*(diam1 / 4 + diam2 / 4) ^3. At the end of treatment, 16 hours after the final dose, plasma samples were taken. Tumours, kidneys and livers removed, weighed, and extracted for paraffin embedding. The study was performed in accordance with UK Home Office regulations under the Animals Scientific Procedures Act 1986 and in accordance with UK National Cancer Research Institute guidelines and the ARRIVE guidelines [21]. Animals were housed in specific pathogen-free rooms in autoclaved, aseptic microisolator cages with a maximum of five animals per cage.

Mice liver and kidney function was assessed by using Aspartate Aminotransferase (AST) Activity Assay Kit (Sigma-Aldrich, Gilligham, UK) and Urea Nitrogen (BUN) Colorimetric Detection Kit (Thermofisher, Waltham, MA, USA) to detect the plasmatic levels of AST and Urea respectively. Data were collected in a Spectrophotometric multiwell plate reader (Perkin-Elmer, Seer Green, Beaconsfield, UK) following manufacturer's protocol.

Supplementary Figure Legend

Supplementary Figure 1. GI50 for cisplatin and gemcitabine. (A) BTC cells were treated with scalar doses of cisplatin (CDDP) or gemcitabine (GEM) for 72 hours. (B) Value of Growth Inhibitory (GI)50 was calculated by using the Prism software. (C) The dose of CDDP and GEM combined (CG) was selected based on the effect on cell viability (<50% compared to DMSO). dCCA: distal CCA; pCCA: perihilar CCA; iCCA: intrahepatic CCA.

Supplementary Figure 2. microRNA expression in human BTC tissues and cell lines. (A) miRNA expression was assessed by Taqman assay in the tumour (TT) and adjacent tissue (AT) of 28 human clinically annotated BTC samples because poor RNA quality was achieved for one AT sample (cohort 1). Bars represent mean values of 2 technical replicates for each patient, expressed as the log of the ratio between TT and AT. (B) Tumour (TT) and adjacent tissue (AT) was extracted from FFPE BTC tissues from cohort 2, N=28). Taqman was used to assess the expression of MIR1249. Bars represent mean values of 2 technical replicates for each patient, expressed as the log of ratio between TT and AT. Blue bars represent the cases with overexpression of MIR1249 >1.3 fold in the tumour tissue. (C) MIR1249 copies were retrieved from RNA-sequencing data of BTC samples in the TCGA dataset. In 9 cases data from tumour and matched non-tumour tissue were available. (D) When the whole TCGA cohort was split according to median tumour MIR1249 expression, median PFI was 264 days in high

MIR1249 vs 549 days in low MIR1249 groups respectively (p: 0.2). However, the whole cohort was highly dyshomogenous including also stage IV disease. Curve represented here are for the TCGA cohort when stage IV cases (because inoperable from diagnosis) and stage III (only one case) were excluded. PFI: Progression Free Interval. E) Data relative to MIR1250 inhibitor in the HTS are provided. Bars represent mean of 3 independent experiments +/- SD. Given the consistent lack of effect in reducing cell viability it was selected as an additional negative control for the validation experiments. (F) Cells were treated with DMSO or CG chemotherapy. After 72 hours same number of cells was collected for RNA extraction. MIR1249 was assessed by Taqman assays. Bars represent mean of three independent experiments +/- SD. (G) Human BTC tissues (N=20 from cohort 3) were stained for CD133 by IHC. Tumours with higher CD133 positivity had higher score for MIR1249 (Chi-square 0.08). (H) CCLP-1 cells were sorted for CD133 surface expression by FACS, cultured in 2D and treated with CG for 72 hours. Dose 1: 3μM C + 30nM G; dose 2: 6μM C + 60nM G). Bars represent mean of six independent experiments +/- SD. (I) CD133+ and CD133- sorted CCLP-1 cells were transfected with the indicated probes for 48 hours and RNA subjected to Taqman assay. Bars represent mean of three independent experiments +/- SD.

Supplementary Figure 3. **Human BTC cell lines with disrupted MIR1249.** CCLP-1 cells were transfected with the pCAS-Guide-EF1a-GFP CRISPR Vector expressing gRNAs for MIR1249. Presence of a lower band (220bp) indicates successful genome editing, while the higher band indicates the WT (280bp). Clone #17 and #56 were selected. (B) Sequences of clones #17 and #56 are aligned to the genomic DNA of the corresponding region, showing the absence of the MIR1249 sequence (highlighted in yellow). (C) Taqman assay was used to verify the lack of MIR1249 expression in the CRISPRed clones #17 and #56. (D) WT and MIR1249KO CCLP-1 cells were analysed for CD133 surface expression by FACS. Bars represent mean of three independent samples +/- SD. On the right representative pictures of the FACS analysis are shown; red dots indicate CD133+ cells. (E) NANOG mRNA expression was assessed by Taqman assay in WT and MIR1249KO cells treated with DMSO and CG. Bars indicate mean +/- SD of three independent experiments. (F) WT and MIR1249KO cells were plated in ULA plates and imaged after 7 days. MIR1249KO cells do not form proper spheroids. (G) MIR1249KO (clone #17) CCLP-1 cells were infected with TripZCTRL or TripZ1249 vector that expresses doxycycline-inducible MIR1249. Cells were activated with doxycycline and treated with DMSO and CG for 72 hours at two concentrations (see above). Bars represent mean of 6 independent experiments +/- SD. (H) CCLP-1 cells were transfected with MIR1249 inhibitor or CTRL inhibitor for 48 hours and treated with the indicated drugs for 72 hours. Bars represent mean of three independent experiments +/- SD. MIR1249 expression was assessed by Taqman. (I) Microarray gene profiling was performed using Agilent two-colour technology. Gene Ontology pathway analysis was run with the Panther enrichment analysis dataset using the differential expressed genes between CTRL-inhibitor transfected cells treated with DMSO or CG (red bars), and the CG-treated cells transfected with CTRL inhibitor or MIR1249 inhibitor (blue bars). Bars represent the % of all pathways. (L) Schematic representation of the binding site for MIR1249 within the 3'-UTR of FZD8 (BiBiServ2 RNAHybrid 2.2; MFE: -36 kcal/mol; $p < 0.0001$). (M) Western blotting for MIR1249KO cell lines with and without transfection with the inducible TripZ vector expressing MIR1249. (N) Representative picture of MIR1249 (ISH) or FZD8 (IHC) staining in human BTC tissue.

Supplementary Figure 4. **In vivo model of CCA treated with weekly i.p. CG.** (A) Mice were injected WT or MIR1249KO CCLP-1 cells subcutaneously (s.c.). At day 14 they were randomized to receive weekly intraperitoneally (i.p.) CG (gemcitabine dose: 150 mg/Kg; cisplatin dose: 2 mg/Kg). Tumour volume was measured by caliper using the following formula 4.19*(Diam1/4+Diam2/4)^3. (B) Representative pictures of kidney and liver explanted from the mice treated with vehicle or CG. Scale bar: 100 μm (C) Plasma was collected from each mouse before sacrifice. AST was measured using the Aspartate Aminotransferase (AST)

Activity Assay kit, while urea measured using the Urea Nitrogen (BUN) Colorimetric Detection kit. Bars represent mean of 10 mice +/- SD.

Supplementary Table 1. Data from the HTS screening. Please see attached pdf file.

Supplementary Table 2. Demographics of cohort 1.

Age		
	median	65
	range	21-76
Gender		
	Female	13
	Male	16
Tumour site		
	iCCA	6
	eCCA	14
	GBC	9
pT		
	1	2
	2	10
	3	10
	4	6
	x	1
pN		
	0	12
	1	15
	x	2
Resection margins		
	R0	23
	R1	5
	Rx	1
Adjuvant chemotherapy		
	no	15
	yes	14

Supplementary Table 3. Demographics of cohort 2. MIR1249 was assessed by ISH in 28 human BTC tissues. MIR1249 expression was classified in negative (0), mild (1+) and strong (2+/3+). Normal liver from patients without BTC were used as controls (N=7) and resulted negative in 4 cases, mildly positive in 2 and 2+positive in 1 case.

Age		
	median	66
	range	44-83
Gender		
	Female	12
	Male	16
Tumour site		
	iCCA	11
	eCCA	7
	GBC	10
pT		
	1	5
	2	9
	3	14
pN		
	0	12
	1	14
	2	1
	x	1
Resection margins		
	R0	24
	R1	3
	R2	1
Size (cm)		
	median	3.8
	range	[0.2-9]
MIR1249 ISH score in BTC		
	0	5
	1+	8
	2+	15
MIR1249 strong / mild or negative		
	iCCA	6 / 5
	eCCA	3 / 4
	GBC	6 / 4

Supplementary Table 4. FZD8 expression is inversely related to MIR1249 expression. Human BTC FFPE tissues were stained for FZD8 expression by IHC and for MIR1249 by ISH. Data of the scoring of the staining are presented here.

	MIR1249 score (ISH)	FZD8 score (IHC)
WT	2+	1+
MIR1249KO	1+	2+
MIR1249KO TripZCTRL	1+	2+
MIR1249KO TripZ1249	2+	0

Supplementary Table 5. Statistical analyses of the "in vivo" experiments. p values indicate ttest.

	day 14	*day 16*	*day 18*	*day 21*	*day 24*	*day 28*	*day 32*	*day 35*	*day 36*
Comparison between MIR1249KO and WT mice									
vehicle-treated xenografts	0.257	0.019	0.362	0.147	0.369	0.157	0.050	0.251	0.156
CG-treated xenografts	0.621	0.367	0.292	0.010	0.181	0.325	0.053	0.067	0.020

References

1. Braconi C, Valeri N, Kogure T, et al. Expression and functional role of a transcribed noncoding RNA with an ultraconserved element in hepatocellular carcinoma. Proc Natl Acad Sci U S A 2011;108:786-91.
2. Carotenuto P, Fassan M, Pandolfo R, et al. Wnt signalling modulates transcribed-ultraconserved regions in hepatobiliary cancers. Gut 2017;66:1268-1277.
